# ResNet based backbone integrated YOLO framework for bone fracture detection

**DOI:** 10.1038/s41598-026-41782-y

**Published:** 2026-03-10

**Authors:** Diptendu Bhattacharya, Subhradip Das, Tamal Biswas, Gouranga Mandal

**Affiliations:** 1 Department of Computer Science and Engineering, National Institute of Technology Agartala, Tripura, India; 2 Department of Computer Science and Engineering, Techno India University, Tripura, India; 3 Manipal Institute of Technology, Manipal Academy of Higher Education, Manipal, India

**Keywords:** Bone abnormality, Bone X-ray, Feature extraction, Fracture detection, ResNet50, YOLO11., Computational biology and bioinformatics, Engineering, Health care, Mathematics and computing

## Abstract

The usage of artificial intelligence and machine learning has significantly strengthened computer-aided medical diagnostics, and fine-tuning models and architectures for medical detection purposes has become a common occurrence. Bone fracture detection is one of the applications where accurate localization of fractures is crucial for proper treatment. In this study, we propose a hybrid ResYOLO11 architecture that combines ResNet50’s feature extraction capability and YOLO11’s detection efficiency in a single model. The proposed architecture uses ResNet layers in the backbone and YOLO11 modules like C3K2, SPPF, and C2PSA to enhance the spatial feature representation, improve the classification precision and detection robustness. The architecture model was trained and evaluated on the public dataset of GRAZPEDWRI-DX, using precision, recall, mAP@50, and mAP@50–95 as performance metrics. The ResYOLO11 architecture achieved precision scores of 0.935, 0.944, 0.945, 0.956, and 0.963, and mAP@50 scores of 0.970, 0.974, 0.977, 0.982, and 0.986 across the nano, small, medium, large, and extra-large variants of the model, respectively. The inference time is 0.012, 0.014, 0.016, 0.019, and 0.026 seconds, respectively, for each model. Quantitative analysis show that ResYOLO11 achieved up to 4.2% higher mAP50 and 6.1% higher mAP50-95 compared to standard YOLO11 variants and was 24% faster in detecting fractures. This comparison showcases the architecture’s potential for assisting orthopedic specialists in accurately identifying fractures and supporting clinical decision-making by providing a clinically robust and computationally efficient solution for computer-aided fracture diagnosis.

## Introduction

With the recent advancement in Artificial Intelligence (AI) and related tools, computer-aided medical diagnostics (CAMD) has been revolutionized. Using AI, machine learning (ML), and deep learning (DL) models to automate CAMD^1^ for the detection and classification of various abnormalities in medical images^2^. DL based techniques, especially convolutional neural networks (CNNs) are used in different models for analyzing X-rays^3^, computed tomography scans (CT scans)^4^, Magnetic resonance images (MRIs)^5^, ultrasounds, etc.

Bone fractures are a significant medical condition impacting people all over the world, across all age groups, impacting life and work^6^. Children and the elderly are the major age groups where fractures are common and hard to treat^7^. Thus, accurate and timely detection is crucial for proper treatment. However, it is very challenging and time-consuming in clinical practice as fractures vary in appearance, and X-ray images are of low contrast. As a result, developing reliable, precise, and automated detection models/architectures has become an active research area of interest in medical image analysis.

Traditional computer vision methods, relying on simple features, have limited detection capability for complex bone fracture structures. Modern DL models for fracture detection prominently use CNN-based frameworks as single-stage detectors such as RetinaNet^8^, CornerNet^9^, CenterNet^10^, SSD^11^, ParallelNet^12^, and You Only Look Once (YOLO)^13^; two-stage models such as R-CNN, Faster R-CNN^14^. Among these YOLO based models and architectures have received significant interest because of its lower size, fast and accurate detection efficiency.

Still, as the standard YOLO architectures primarily focuses on detection efficiency and not accuracy which is crucial in CAMD. So, to address this limitation, we integrated residual networks (ResNet) into YOLO architecture enhancing the representation by preserving gradient flow and capturing multi-scale spatial features. Motivated by this, the proposed ResYOLO11 model uses customized ResNet50 in the backbone of YOLO11 model with C3K2, SPPF, and C2PSA modules, ensuring spatial management and efficiency of YOLO.

The key contributions of this study are summarized as follows:

1. A novel ResYOLO11 architecture integrating custom ResNet50 based backbone with YOLO11’s neck and head to enhance feature extraction for bone fracture detection.
2. A comprehensive preprocessing pipeline involving traditional noise reduction and contrast enhancement to improve data quality before model training.
3. Extensive experimentation on the GRAZPEDWRI-DX dataset, with cross-validation to ensure robustness and generalization.
4. Detailed performance comparison with multiple YOLO variants, demonstrating superior precision, recall, and mean average precision (mAP) scores while reducing model size and inference time.

The structure of this paper is organized as follows: Section “Literature review” reviews related literature on fracture detection and object detection architectures. Section “Proposed methodology” presents the proposed methodology, including dataset, preprocessing, and proposed model architecture. Section “Experiments” discusses the experimental process and ablation studies. “Results and Discussion” discusses the experimental results, comparative analyses, and performance implications. Section “Conclusion” concludes the paper with potential directions for future work.

## Literature review

Bone fracture detection from X-ray images is an active research area. With the development of ML and DL for CAMD, the efficiency for accurate detection has seen major growth. The fracture detection process mainly consists of two major stages: (i) first, the preprocessing of images for enhancement and normalization, (ii) second, the main detection or classification architecture/model.

### Preprocessing

Image preprocessing is an important step for ensuring image quality and uniformity of size across the dataset before training^15^. Standard preprocessing steps include channel adjustment^16^, cropping, resizing the images to fit the model requirements^17^, noise removal, and enhancement of images^18^.

Numerous filtering techniques have been developed for reducing noise as well as enhancing the quality of X-ray images by edge-preservation. Median filter^19^ is the most popular and generalized artifact noise removal filter. The median filter removes salt and pepper noise by replacing noisy pixels with the median value of their neighborhood. Similar to the median filter, linear filters also replace noisy pixels with the mean value of neighboring pixels^20^. This helps to suppress the additive noise in images. The non-linear filter uses morphological and statistical transformations to preserve structure and remove noise^21^. Adaptive filters are dynamic filters that change filtering parameters based on local image characteristics, improving denoising and image restoration in heterogeneous regions^22^. Anisotropic diffusion filters further refine this process by diffusing noise while maintaining sharp edges^23^. CNNs and auto encoders are also gaining popularity in noise removal, leveraging DL models^24^.

**Table 1:** Filtering methods for image processing.

Author	Method	Description
^19^	Median filter	Changes the pixel value with the median of its neighborhood.
		Effective for salt-and-pepper noise.
^20^	Linear filter	Changes the pixel value with the average of its neighborhood.
		Effective for linear noise.
^21^	Non-linear filter	Uses non-linear operations like morphological filtering to reduce noise.
		It preserves the edges while filtering.
^22^	Adaptive filter	Based on the image characteristics, it adjusts the filter parameters.
		It is effective for denoising, enhancement, and image restoration.
^23^	Anisotropic diffusion filter	Uses a constant diffusion coefficient to reduce the noise by diffusing it in the surroundings.
		It smoothens the image without blurring the edges.

Some of the techniques of noise removal are discussed in the Table 1.

### Detection

Bone fracture detection methods using DL models can be broadly categorized as two-stage and one-stage architectures. In two-stage detectors, regions of interest are detected first, and later classification is done based on them^25^; whereas in one-stage detection, detection and classification go hand-in-hand^26^.

A study on a two-stage fracture detection model^27^ proposed Faster R-CNN with CrackNet as a model to detect fractures from a custom dataset of skull, trunk, and limb fractures. The model obtained a precision of 89% for fracture detection. Another study used the MURA dataset to perform one-stage and two-stage detection for bone fractures^28^, where the average sensitivity achieved was of 61.53% and 70.04% and the specificity was 84.22% and 74.76%, respectively. A similar study was conducted on pelvic CT scans using symmetry as a reference to detect fractures^29^. The proposed model resulted in an average precision of 92%. Also, another approach using two-stage detection used a convolutional neural network for classifying and YOLOv8 achieved mAP50 of 93.5 for cervical spine fracture detection^30^.

For one-stage detection methods, a review was performed to find different methods such as YOLO and Single Shot Multibox Detector (SSD) and explained their applications^31^. A YOLOv7 model was proposed for full-body fracture detection^32^ using the FracAtlas dataset and obtained mAP50 of 86.2%. YOLOv8 was also used for elbow fracture detection which obtained a F1 score of 78.1% and mAP50-95 of 78.7%, as proposed in^33^. A large dataset was published for wrist fracture detection^34^, called “GRAZPEDWRI-DX”, where the authors presented baseline results with YOLOv5 at mAP50 of 0.93 for fracture class.

Similar researches were conducted with the help of YOLOv4^35^ which achieved mAP50 of 89%,, YOLOv5^36^ with mAP50 0.69, YOLOv6^36^ with mAP50 0.64, and YOLOv7^36^ with mAP50 0.628 for ‘all’ (macro-average across classes) classes, YOLOv8^36^ with mAP50 0.77 for all classes on the GRAZPEDWRI-DX dataset.

A summary of recent YOLO-based models for wrist bone fracture detection is presented in Table 2, where ‘All classes’ refers to the macro average over all annotation classes. The table illustrates the progress in the enhancement of Detection performance across generations of YOLO. Even with these improvements, the existing YOLO frameworks are limited by feature extraction from noisy and low-contrast X-ray images. This is the main motivation behind the integration of ResNet into the YOLO architecture to enhance detection and localization of fractures.

**Table 2:** Performance analysis (mAP@50) of YOLO models for wrist trauma detection.

Author	Model	Fracture	All classes
^35^	YOLOv4	0.89	–
^34^	YOLOv5	0.933	0.622
^36^	YOLOv5x	0.95	0.69
^36^	YOLOv6m	0.94	0.64
^36^	YOLOv7	0.94	0.61
^36^	YOLOv8x	0.95	0.77
^37^	YOLOv9-E	–	0.654
^38^	YOLOv9+Aug1	–	0.612
^39^	YOLOv10	–	0.709

### Existing YOLO hybrids

Recently, hybrid approaches integrating the efficiency of YOLO models with ResNet and other DL methods have been developed. One of the prominent approaches includes the proposal of a two-stage classifier-detection approach^40^, where ResNet50 was used for classification and YOLOv7 for detection. The validation was conducted on a custom dataset integrating the ‘Stanford Mura Dataset’, ‘Fracatlas Dataset’, and ‘GRAZPEDWRI-DX Dataset’. The model achieved an accuracy score of 0.977 for classification and mAP50 of 0.824 for fracture localization.

In another study, an EfficientDet-YOLO NAS (v8 inspired)^41^ was used to detect fractures from a combined dataset, which contained 4736 images and achieved 0.989 in precision and 0.991 in mAP50. ResNet, SeResNet, and ViT was also used to hybridize YOLOv8^42^, in another approach where YOLO feature extraction was used with ResNet, SeResNet, and ViT based detector. The model achieved a staggering 99% classification accuracy on the detected region of interest proposed by the YOLOv8 feature extractor.

A CNN based YOLO^43^ model was introduced which scored an average accuracy of 79.5% for fracture classification. Depth-wise convolution was also formulated in another approach and replaced Convolution modules in the YOLO architecture of DWYOLO^44^ achieving mAP50 of 0.889 for fracture identification. Other significant YOLO hybridizing approaches include using any YOLO model (eg. YOLOv10) for detection followed by another DL based transfer learning architecture of EfficientNet, ResNet, DenseNet, or GoogleNet, etc.^45^

## Proposed methodology

**Fig. 1:**
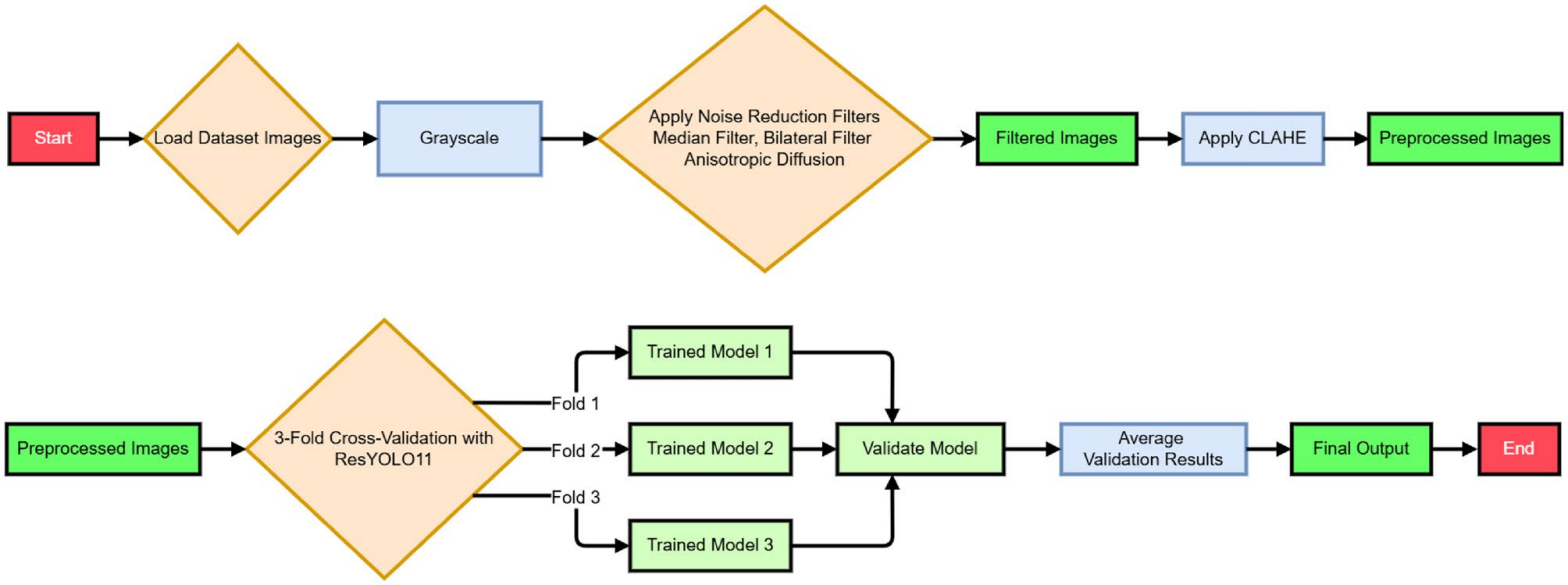
Proposed training and validating workflow.

The proposed methodology for the study comprises dataset description and splitting, preprocessing, and enhancement, the proposed ResYOLO11 architecture, training setup, and evaluation metrics. A detailed workflow diagram is shown in the Fig. 1.

### Dataset

The proposed model is trained and validated using the public dataset “GRAZPEDWRI-DX”^34^. The dataset was published by the Department for Pediatric Surgery of the University Hospital Graz, based on the data of 6091 patients with wrist trauma between 2008-2018. It contains 10,643 studies with 20,327 X-ray images, annotated with 67,771 labeled objects. The classes include ‘text’, ‘fracture’, ‘bone anomaly’, ‘bone lesion’, ‘foreign body’, ‘metal inclusion’, ‘periosteal reaction’, ‘pronator sign’, and ‘soft tissue’.

The dataset was chosen due to its high clinical diversity, standard labeling, and prior benchmarks across older YOLO variants, thus is helpful for proper comparative analysis.

3-fold cross-validation was employed to ensure the generalization of the model and detect overfitting. Each fold divided the dataset into 2:1 train-validation set ratio, resulting in 13,552, 13,551, and 13,551 images for training and 6775, 6776, and 6776 images for validation, respectively.

The distribution of data across various annotations in each fold is shown in Table 3. Although internally each fold suffers from class imbalance for rarer classes, overall each fold is balanced. Moreover the imbalance acts a means to simulate the actual probability of these rare situations. Also, the main objective of the study is to improve bone fracture detection, using the dataset as it is distributed is justified.

**Table 3:** Abnormality statistics in the dataset.

**Class**	**Fold0**		**Fold1**		**Fold2**	
	**Images**	**Instances**	**Images**	**Instances**	**Images**	**Instances**
All	6775	15900	6776	15833	6776	15710
Boneanomaly	64	102	71	95	57	79
Bonelession	13	13	17	18	12	14
Foreignbody	2	2	4	4	2	2
Fracture	4579	6103	4500	5999	4471	5988
Metal	227	268	253	285	227	265
Periostealreaction	756	1179	761	1159	718	1115
Pronatorsign	208	209	175	175	183	183
Softtissue	151	162	134	143	154	159
Text	6756	7862	6763	7955	6755	7905

### Preprocessing and enhancement

As X-ray images in the dataset often suffer from low contrast, non-uniformity of size, and noise artifacts, this makes accurate feature extraction difficult. Thus, to improve the input data quality, a multi-stage pipeline was applied before model training.

1. Size: First, all the input images were normalized to have 640p size.
2. Noise: For noise reduction, multiple filters were used while retaining edge and texture details. The filters include:
  - Median Filter was used to remove overall impulse (salt-and-pepper) noise.
  - Bilateral Filter was next, as it performs edge-preserving smoothing by combining spatial and intensity similarity functions.
  - Then, an Anisotropic Diffusion Filter was used to reduce high-frequency noise by iteratively diffusing intensity values while maintaining the structural edges.
3. Contrast: After denoising, Contrast Limited Adaptive Histogram Equalization (CLAHE) was applied to improve local contrast and emphasize bone boundaries, thereby enhancing fracture visibility.

An ablation study was conducted with YOLO11n as the model with the same hyperparameters on a 2000 image subset of the dataset. The image size ablation indicates that with increasing input size the detection accuracy of single stage detectors improves, but this improvement comes at the cost of memory and inference time. The ablation results are shown in Table 4 where, While 1024 $\times$ 1024 achieves marginally higher mAP values, it results in a twofold increase in inference latency with limited recall improvement. In contrast, 640 $\times$ 640 provides the best balance between precision, recall, and computational efficiency, making it more suitable for real-time and clinical deployment. Therefore, 640 $\times$ 640 was selected as the default input resolution for all subsequent experiments. Furthermore using 1024p to train and validate the larger variants of YOLO11 and ResYOLO11 is not feasible in the current study and hence noted for future work. Similarly, another ablation study was conducted for verifying the impact of various enhancement and filters in fracture detection. The relevant data is shown in Table 5. The table showcases consistent incremental improvements as additional preprocessing steps were introduced. Median and bilateral filtering improve robustness of the model against noise while preserving fracture edges, anisotropic diffusion enhances the structural consistency and localization accuracy. Finally, CLAHE increases local contrast, leading to the highest precision, recall, and mAP scores. These results justify the inclusion of all enhancement components in the final preprocessing pipeline.

**Table 4:** Ablation study analyzing the impact of input image resolution on fracture detection performance.

Image size	Precision	Recall	mAP@50	mAP@50–95	Inference time (s)
320 $\times$ 320	0.641	0.772	0.792	0.401	0.0005
640 $\times$ 640	0.747	0.829	0.840	0.451	0.001
1024 $\times$ 1024	0.791	0.793	0.845	0.455	0.002

**Table 5:** Ablation study evaluating the effect of progressive image enhancement techniques on fracture detection performance.

Enhancement	Precision	Recall	mAP@50	mAP@50–95
A: No enhancement	0.747	0.829	0.840	0.451
B: Median filter	0.754	0.832	0.843	0.454
C: B + Bilateral filter	0.762	0.836	0.847	0.458
D: C + Anisotropic diffusion	0.769	0.841	0.851	0.463
E: D + CLAHE (full pipeline)	0.781	0.848	0.856	0.469

**Fig. 2:**
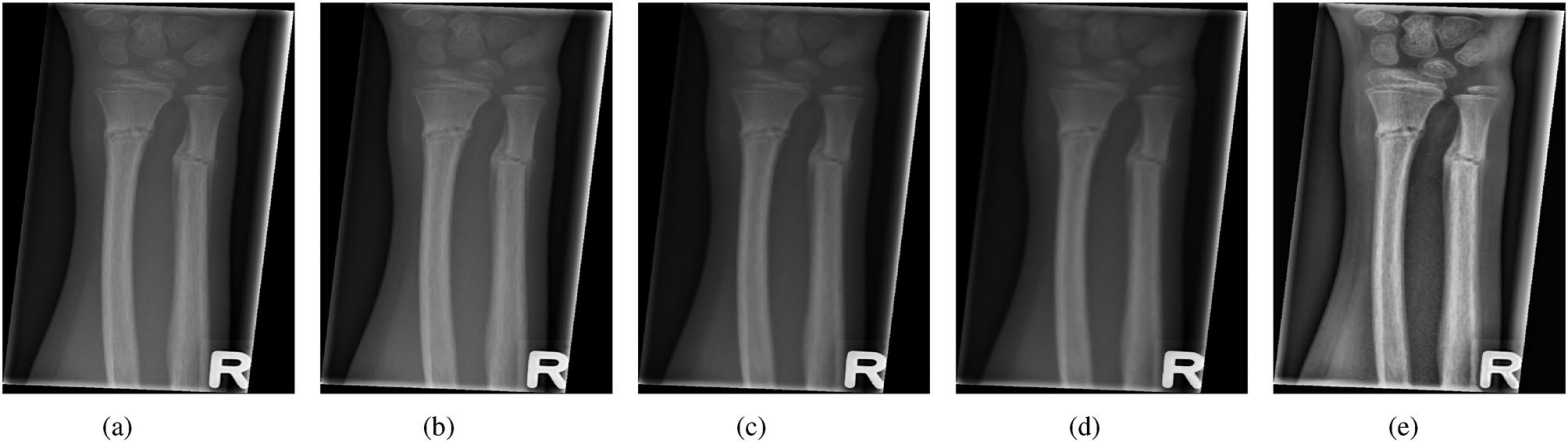
Image enhancement results applied to X-ray Images.

The combined effect of these steps is shown in Fig. 2 where, the Fig. 2a shows the original X-ray image from the dataset, Fig. 2b shows the median filtered version, Fig. 2c is the state of the input data after application of the bilateral filter, anisotropic diffusion is shown in Fig. 2d, and the CLAHE-enhanced image is shown in Fig. 2e.

### ResYOLO11 architecture

The proposed ResYolo11 architecture integrates residual feature extracting capability of ResNet50 with the detection efficiency of YOLO11^46^ to produce a hybrid one-stage detection model optimized for classification and localization of fractures. The proposed model uses a customization on the original ResNet structure to make it YOLO compatible.

The main features of the proposed architecture include:

#### Backbone

The backbone of the proposed model consists of four ResNet modules for extracting hierarchical features from input images and a convolution block for further refinement of the features. The ResNet module comprises 2-3 convolution layers with residual (skip) connections. This helps gradients to flow more easily through the network, especially during back propagation, and addresses the vanishing gradient problem of multi-layered DL models.

#### Neck

The neck of the proposed ResYOLO11 model is responsible for multi-scale feature aggregation from the backbone. The section uses several original YOLO11 modules for ease of computation and spatial management. The modules include:

1. C3k2 (Cross Stage Partial block) replaces the traditional C2f bottlenecks, mainly for reducing computational demands while maintaining feature quality. C3K2 blocks incorporate partial dense connections and bottleneck operations for efficient processing.
2. SPPF (Spatial Pyramid Pooling – Fast) aggregates (pools) contextual information across different scales using different kernel sizes for improving prediction robustness. It is derived from SPP (Spatial Pyramid Pooling) which was used in older YOLO versions. SPPF preserves spatial dimensions and is highly efficient, and avoids fixed-length output vectors.
3. C2PSA Cross Stage Partial with Spatial Attention) extends CSP by incorporating spatial attention (PSA: Parallel Spatial Attention). The module splits the input, applies pointwise convolutions, and integrates an attention mechanism that lets the network focus on relevant regions in the feature map dynamically. This improves the discriminative capability of the model, making the network more sensitive to important image regions by pooling and weighting features spatially.
4. Upsample-Concat modules are used for resizing the feature maps to larger spatial sizes, and for effective fusion of features from different sizes. By working together, both modules enable multi-scale feature aggregation required for detecting objects of varying sizes.

#### Head

The refined output from the neck is passed to three Detection layers (block 20), each responsible for predicting bounding boxes and class probabilities for different scales/features maps. These multi-scale aggregated features are processed with additional convolutional (CBS: Conv-BatchNorm-SiLU) blocks to further refine information.

**Fig. 3:**
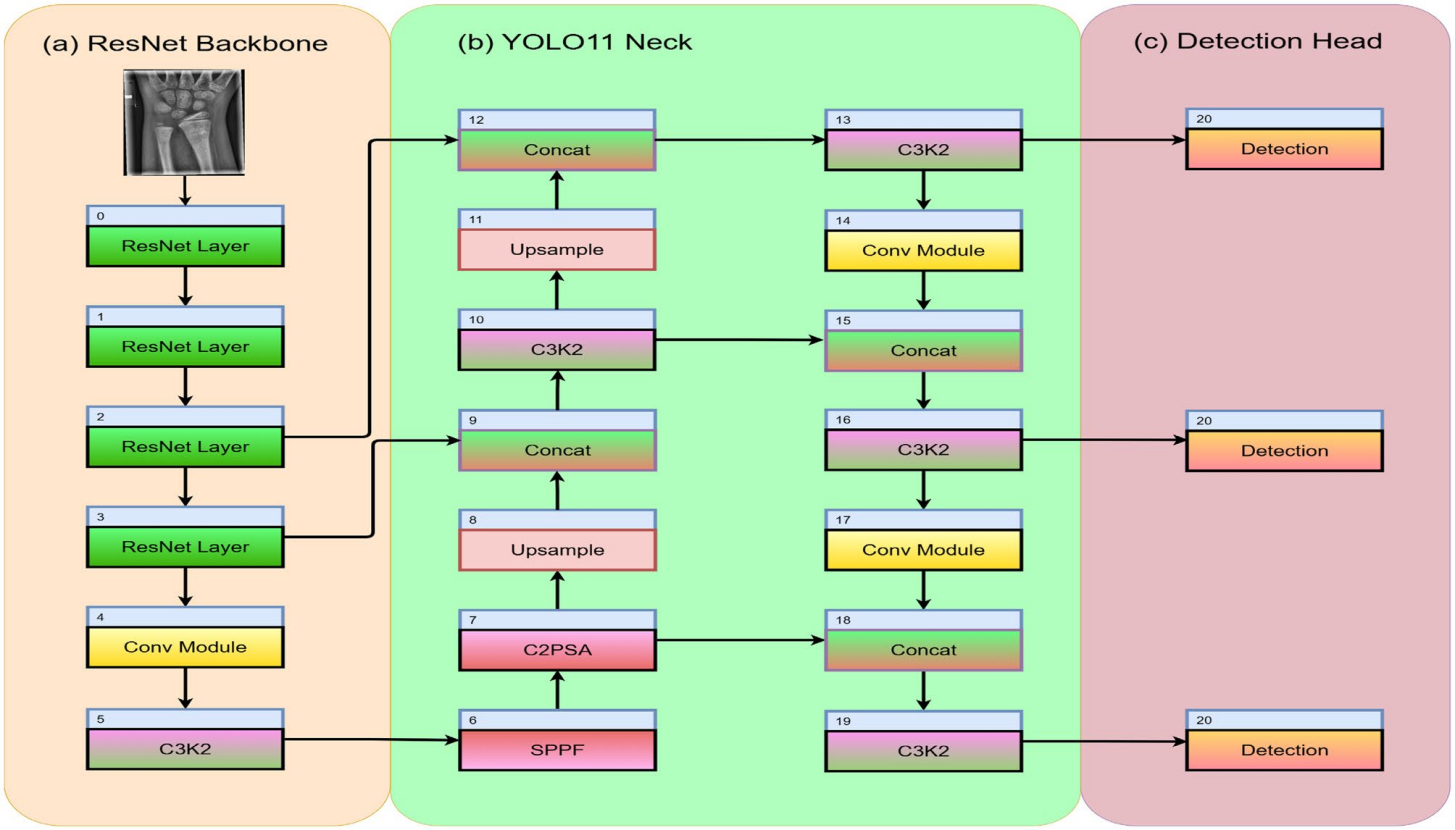
Model Architecture of ResYOLO11.

The resulting architecture consists of approximately 388–589 layers, depending on the variant (nano to extra-large). This design balances representational depth and computational efficiency, achieving improved mAP and reduced inference latency compared to baseline YOLO11 models. The complete architecture is shown in Fig. 3.

### Model justification

The proposed ResYOLO11 architecture design is motivated by the fundamental limitations of standard YOLO models and is a targeted enhancement to specifically adapt to the challenges of bone fracture. As compared to natural images, X-ray images often exhibit low contrast, noise artifacts, and subtle structural variations, making reliable feature extraction difficult. Furthermore, the standard backbone is primarily optimized for natural images and may not sufficiently capture the fine-grained details required for accurate fracture localization. To address this limitation, residual learning is integrated into the YOLO11 backbone, resulting in the proposed ResYOLO11 architecture, which is still a single-stage detector leveraging ResNet feature extraction.

The residual connections allow features features from earlier layers to be reused again in deeper layers, thus improving the gradient flow during training. It also allows the network to remember minute structural information from the original image. The ResNet modules are arranged sequentially in the backbone so that increasing depth enhances the model’s representational capacity in a controlled manner.The integration of ResNet blocks within YOLO11 is further justified by their compatibility with YOLO11’s neck and head design. While ResNet strengthens the backbone’s discriminative feature extraction, YOLO11’s lightweight neck−comprising C3K2, SPPF, and C2PSA modules−ensures efficient multi-scale feature aggregation and spatial attention. In particular:

- C3K2 modules reduce computational overhead while maintaining feature richness through partial dense connectivity.
- SPPF modules enhance contextual awareness by aggregating multi-scale receptive fields without increasing inference latency.
- C2PSA modules introduce spatial attention, enabling the network to selectively emphasize anatomically relevant regions such as fracture lines, and periosteal reactions.

The most important design consideration of the proposed model is that it retains its status and benefits as a single-stage detector. Unlike two-stage hybrids^40^ or cascade-based approaches, the proposed method does not separate between feature extraction, classification, or detection nor does it require separate refinement stages. This in return keeps the inference process simple and fast, which is crucial for practical clinical use.

## Experiments

### Training strategy

For the evaluation and design of the architecture, all models were trained on **NVIDIA Tesla T4 GPUs (15 GB VRAM each)** (Kaggle) using the Stochastic Gradient Descent (**SGD**) optimizer with a **learning rate** of **0.01**, **momentum** of **0.9**, and **weight decay** of **0.0005**. The input **image size** was normalized at **640 **$\times$ **640 pixels**, the **batch size** at **32**, and training was conducted for **100 epochs per fold**.

During each training cycle, early stopping and learning rate scheduling were employed to prevent overfitting. The final model performance was averaged across the three validation folds. Each ResYOLO11 variant (nano, small, medium, large, extra-large) was compared with its corresponding YOLO11 baseline for an equitable evaluation of scalability.

### Evaluation metrics

The evaluation and comparison of our model ResYOLO11 with the YOLO11 model, was conducted on the basis of object detection metrics. Considering, Tr Po = True Positive, Fl Po = False Positive, Fl Ne = False Negative, i = ith Object Class, and N = Number of All Classes, a short description of the metrics are as follows:

- Precision (Pre): For measuring the model accuracy in object identification, among all predicted detections. A high precision value is an indication of the fact that the model gives fewer false alarms, meaning that the bounding boxes predicted are more likely to be correct. It is expressed as Eq. 1:
  1 $$\begin{aligned} Pre = \frac{Tr\ Po}{ Tr\ Po\ +\ Fl\ Po } \end{aligned}$$
- Recall (Rec): To evaluate the model’s detection ability for all objects present in the actual data. A high recall indicates that the model is capable of identifying most of the objects present in the image, thereby minimizing missed detections. It is expressed as Equation 2:
  2 $$\begin{aligned} Rec = \frac{Tr\ Po}{Tr\ Po\ +\ Fl\ Ne} \end{aligned}$$
- Mean Average Precision (mAP): For evaluating both detection accuracy and localization performance of the model across all object classes. It is given by Equation 3:
  3 $$\begin{aligned} \text {mAP} = \frac{1}{N} \sum _{i=1}^{N} \text {AP}_i \end{aligned}$$
  Average Precision (AP): It is the area under the precision-recall curve which is calculated for each class. It is given by Equation 4:
  4 $$\begin{aligned} \text {AP} = \int _{0}^{1} pre(rec) \, dr \end{aligned}$$

The rationale behind choosing these metrics is that there are most widely used metrics and evaluates object detection models in all required criteria. Furthermore, using the harmonic mean of Pre and Rec F1 score (F1) can easily be obtained and mAP is advanced form of the traditional IoU metric. mAP simultaneously evaluate localization accuracy and classification confidence across IoU thresholds and thus is inclusive of it.

### Ablation study

To better understand the contribution of individual architectural components and design choices, an ablation study was conducted on the proposed ResYOLO11 model. The objective of this analysis is to quantify the impact of residual feature extraction and YOLO11 neck modules on fracture detection performance, and to demonstrate that the observed improvements are not solely due to increased model depth or parameter count.

For the ablation study, 4000 images (nearly 20%) of the GRAZPEDWRI-DX dataset was used to ensure a controlled and computationally efficient evaluation of architectural components. All experiments were performed using identical training settings, including image resolution, optimizer, learning rate, and number of epochs. The ResYOLO11-m variant was selected as the reference model to ensure a balanced evaluation between performance and computational cost. The comparison was done on the basis of fracture class only.

**Table 6:** Ablation study showing the impact of different ResNet backbones integrated into YOLO11-m on the GRAZPEDWRI-DX dataset.

Model	Precision	Recall	mAP@50	mAP@50–95	Size (MB)	Parameters	Inference time (s)
YOLO11m	0.614	0.791	0.806	0.414	40.5	20,059,947	0.013
ResNet-18	0.602	0.794	0.787	0.401	40.6	20,097,579	0.015
ResNet-34	0.602	0.787	0.794	0.402	40.4	20,001,835	0.016
ResNet-50	0.702	0.803	0.796	0.407	47.9	23,746,603	0.017
ResNet-101	0.668	0.751	0.776	0.396	83.9	41,621,547	0.026
ResNet-152	0.571	0.746	0.784	0.391	106.3	52,793,387	0.033

The ablation results in Table 6 indicate that integrating residual learning improves detection performance up to an optimal depth. While shallow backbones like ResNet-18 and ResNet-34 provide limited gains, ResNet-50 achieves the best balance between precision, recall, and localization accuracy. Also, further increasing the depth to ResNet-101 and ResNet-152 leads to performance degradation despite higher computational cost, suggesting over-parameterization and reduced feature generalization for X-ray fracture detection. These findings justify the selection of ResNet-50 as the backbone in the final ResYOLO11 architecture.

## Results and discussion

The experimental results obtained from training and validation of the proposed ResYOLO11 architecture on the ‘GRAZPEDWRI-DX’ dataset is presented in this section, followed by analytical discussion and comparison with baseline YOLO11 models across nano, small, medium, large, and extra-large variants. Results are averaged across 3 folds

### Quantitative evaluation

From Table 7, it is clear that ResYOLO11 achieved higher precision scores across all the model variants, compared to YOLO11. Furthermore, for fractures the highest precision was obtained by ResYOLO11x (0.963) and across all variants the scores differed from 0.934 to 0.963, increaseing with model size for the proposed model; while YOLO11’s precision ranged from 0.896 to 0.939 with the order of performance being $m>l>n>x>s$.

**Table 7:** Precision comparison between RESYOLO11 and YOLO11 models.

**Precision**	**RESYOLO11**					**YOLO11**				
	**n**	**s**	**m**	**l**	**x**	**n**	**s**	**m**	**l**	**x**
**All**	**0.829**	**0.837**	**0.830**	**0.835**	**0.864**	**0.820**	**0.774**	**0.813**	**0.810**	**0.795**
	±0.021	±0.020	±0.016	±0.066	±0.019	±0.036	±0.047	±0.049	±0.080	±0.007
Boneanomaly	0.779	0.831	0.783	0.777	0.856	0.762	0.624	0.759	0.786	0.779
Bonelession	0.758	0.824	0.770	0.838	0.809	0.764	0.779	0.728	0.753	0.713
Foreignbody	1.000	1.000	1.000	0.966	1.000	1.000	0.995	1.000	0.922	0.772
**Fracture**	**0.934**	**0.944**	**0.945**	**0.956**	**0.963**	**0.930**	**0.896**	**0.939**	**0.931**	**0.927**
	±0.005	±0.003	±0.008	±0.036	±0.011	±0.021	±0.015	±0.012	±0.027	±0.009
Metal	0.911	0.914	0.917	0.928	0.972	0.938	0.893	0.921	0.941	0.936
Periostealreaction	0.742	0.739	0.746	0.723	0.811	0.719	0.629	0.718	0.735	0.695
Pronatorsign	0.696	0.684	0.704	0.701	0.718	0.671	0.613	0.654	0.676	0.686
Softtissue	0.668	0.623	0.628	0.654	0.670	0.622	0.572	0.619	0.578	0.673
Text	0.974	0.975	0.977	0.974	0.980	0.971	0.963	0.976	0.970	0.971

Bolded rows represent the main categories emphasized for comparison in this table.

A similar pattern is seen in Table 8, where ResYOLO11 performed exceptionally well for recall scores. Though the maximum recall for fracture was obtained by YOLO11l (0.960) where ResYOLO11l scored 0.957, the average recall across all clases was higher in ResYOLO11, indicating overall reliability for all classes.

**Table 8:** Recall comparison between RESYOLO11 and YOLO11 models.

**Recall**	**RESYOLO11**					**YOLO11**				
	**n**	**s**	**m**	**l**	**x**	**n**	**s**	**m**	**l**	**x**
**All**	**0.693**	**0.708**	**0.675**	**0.821**	**0.770**	**0.682**	**0.709**	**0.664**	**0.782**	**0.736**
	±0.037	±0.012	±0.022	±0.068	±0.015	±0.039	±0.079	±0.051	±0.132	±0.027
Boneanomaly	0.459	0.470	0.590	0.703	0.739	0.482	0.492	0.575	0.700	0.578
Bonelession	0.444	0.479	0.573	0.768	0.677	0.406	0.540	0.585	0.761	0.685
Foreignbody	0.483	0.583	0.000	0.650	0.417	0.358	0.550	0.000	0.326	0.354
**Fracture**	**0.940**	**0.939**	**0.945**	**0.957**	**0.958**	**0.927**	**0.944**	**0.939**	**0.960**	**0.953**
	±0.036	±0.062	±0.103	±0.045	±0.124	±0.097	±0.058	±0.069	±0.080	±0.173
Metal	0.952	0.959	0.961	0.975	0.973	0.946	0.963	0.955	0.968	0.971
Periostealreaction	0.681	0.715	0.703	0.852	0.730	0.665	0.620	0.699	0.799	0.774
Pronatorsign	0.825	0.772	0.799	0.859	0.840	0.781	0.754	0.701	0.834	0.812
Softtissue	0.476	0.478	0.526	0.638	0.617	0.591	0.534	0.536	0.701	0.514
Text	0.979	0.980	0.981	0.985	0.981	0.982	0.985	0.983	0.985	0.985

Bolded rows represent the main categories emphasized for comparison in this table.

For mAP two categories were calculated, Table 9 for mAP at threshold 50, and Table 10 for mAP50-95. The tables show that ResYOLO11 architecture achieved higher detectionaccuracy across all IoU thresholds. For mAP50, the values improved from 0.968 (YOLO11n) to 0.970 (ResYOLO11n) and from 0.981 (YOLO11x) to 0.986 (ResYOLO11x). Similarly, the mAP50–95 increased from 0.643 to 0.661 for YOLO11n and ResYOLO11n, with the highest being 0.728 for ResYOLO11x, marking the consistent improvement with model depth.

**Table 9:** mAP50 comparison between RESYOLO11 and YOLO11 models.

**mAP50**	**RESYOLO11**					**YOLO11**				
	**n**	**s**	**m**	**l**	**x**	**n**	**s**	**m**	**l**	**x**
**All**	**0.719**	**0.727**	**0.740**	**0.803**	**0.824**	**0.701**	**0.731**	**0.736**	**0.798**	**0.781**
	±0.016	±0.014	±0.002	±0.031	±0.038	±0.007	±0.045	±0.024	±0.037	±0.021
Boneanomaly	0.554	0.658	0.659	0.651	0.875	0.584	0.541	0.668	0.733	0.735
Bonelession	0.431	0.610	0.649	0.760	0.778	0.461	0.608	0.659	0.729	0.690
F	0.411	0.241	0.259	0.670	0.452	0.175	0.402	0.342	0.427	0.522
**Fracture**	**0.970**	**0.974**	**0.977**	**0.982**	**0.986**	**0.968**	**0.970**	**0.973**	**0.978**	**0.981**
	±0.017	±0.031	±0.097	±0.106	±0.096	±0.070	±0.074	±0.099	±0.045	±0.169
Metal	0.971	0.977	0.979	0.984	0.982	0.977	0.976	0.979	0.986	0.985
Periostealreaction	0.745	0.752	0.768	0.790	0.822	0.731	0.748	0.716	0.813	0.787
Pronatorsign	0.817	0.812	0.820	0.780	0.857	0.792	0.797	0.799	0.826	0.810
Softtissue	0.578	0.531	0.561	0.621	0.673	0.628	0.548	0.497	0.698	0.525
Text	0.991	0.991	0.992	0.992	0.992	0.992	0.992	0.992	0.992	0.992

Bolded rows represent the main categories emphasized for comparison in this table.

**Table 10:** mAP50-95 comparison between RESYOLO11 and YOLO11 models.

**mAP50-95**	**RESYOLO11**					**YOLO11**				
	**n**	**s**	**m**	**l**	**x**	**n**	**s**	**m**	**l**	**x**
**All**	**0.480**	**0.498**	**0.521**	**0.545**	**0.598**	**0.481**	**0.484**	**0.514**	**0.542**	**0.536**
	±0.018	±0.015	±0.015	±0.023	±0.032	±0.009	±0.041	±0.026	±0.020	±0.020
Boneanomaly	0.329	0.459	0.424	0.364	0.545	0.357	0.324	0.400	0.473	0.404
Bonelession	0.327	0.413	0.474	0.564	0.580	0.325	0.415	0.472	0.572	0.500
Foreignbody	0.195	0.130	0.180	0.364	0.303	0.119	0.139	0.156	0.275	0.297
**Fracture**	**0.661**	**0.671**	**0.683**	**0.688**	**0.728**	**0.643**	**0.643**	**0.668**	**0.704**	**0.675**
	±0.025	±0.035	±0.063	±0.047	±0.078	±0.040	±0.031	±0.107	±0.054	±0.143
Metal	0.800	0.799	0.842	0.863	0.897	0.862	0.852	0.804	0.696	0.881
Periostealreaction	0.403	0.416	0.435	0.447	0.506	0.384	0.399	0.440	0.488	0.457
Pronatorsign	0.498	0.511	0.527	0.496	0.584	0.493	0.493	0.539	0.547	0.516
Softtissue	0.363	0.328	0.370	0.370	0.483	0.406	0.337	0.397	0.365	0.336
Text	0.744	0.751	0.753	0.746	0.756	0.746	0.751	0.747	0.757	0.756

Bolded rows represent the main categories emphasized for comparison in this table.

Also because of natural class imbalance, classes like foreignbody, bonelession, etc suffer from low scores in those metrics, thereby impacting performance of the models.

**Fig. 4:**
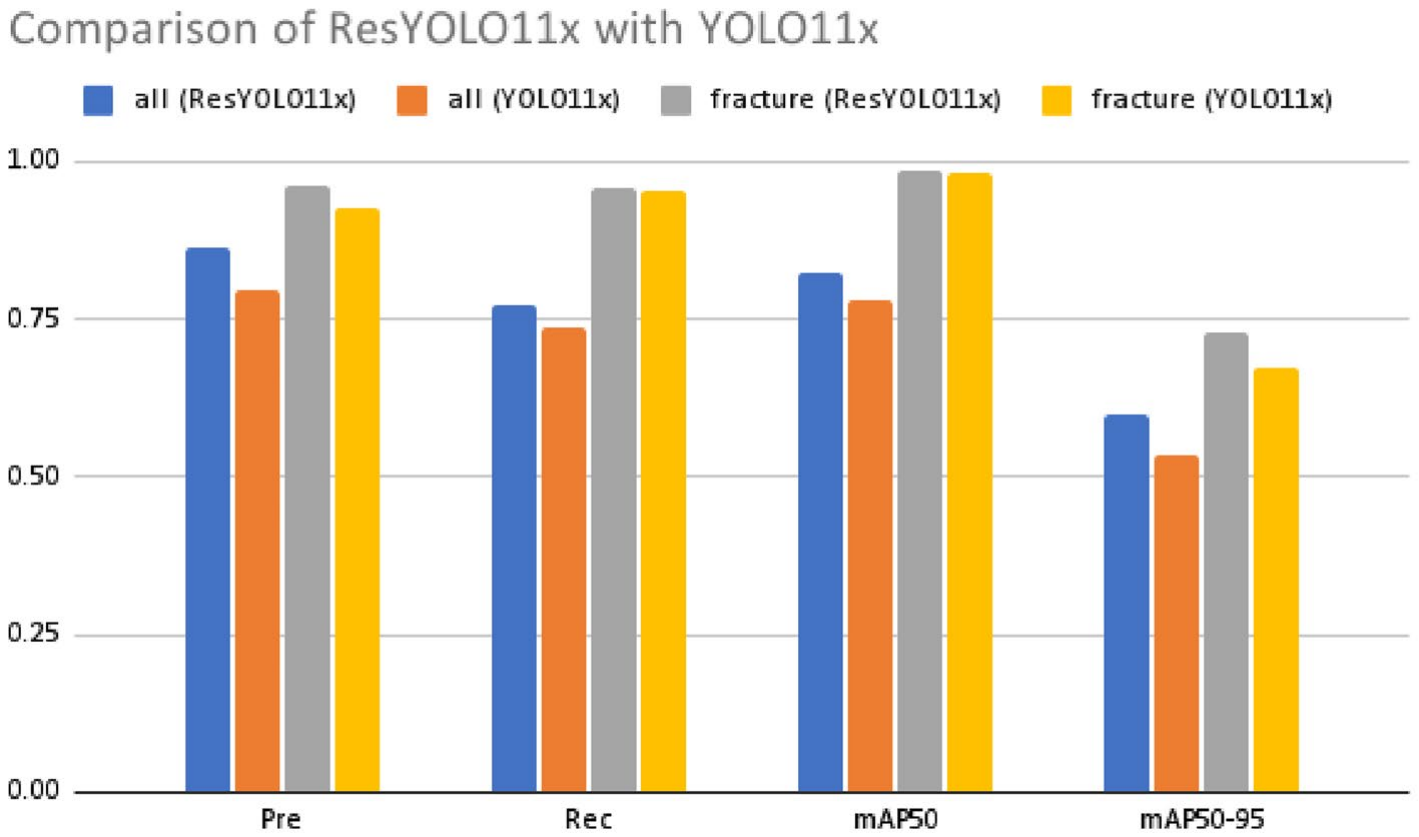
Comparision of ResYOLO11x with YOLO11x.

A graphical comparison of the results for the applied matrices of our model is shown in Fig. 4. As seen from the graph, in both ”all class” and ”Fracture”, the ResYOLO11x model performs consistently higher in precision. Next, comparable improvements are seen in mAP50 and mAP50-95. Moreover from Table 7, it is clear that using ResNet layers in the model greatly increases the precision of the model, and from Tables 8,9, and 10, there is an increase in performance compared to YOLO11 models.

### Qualitative evaluation

**Fig. 5:**
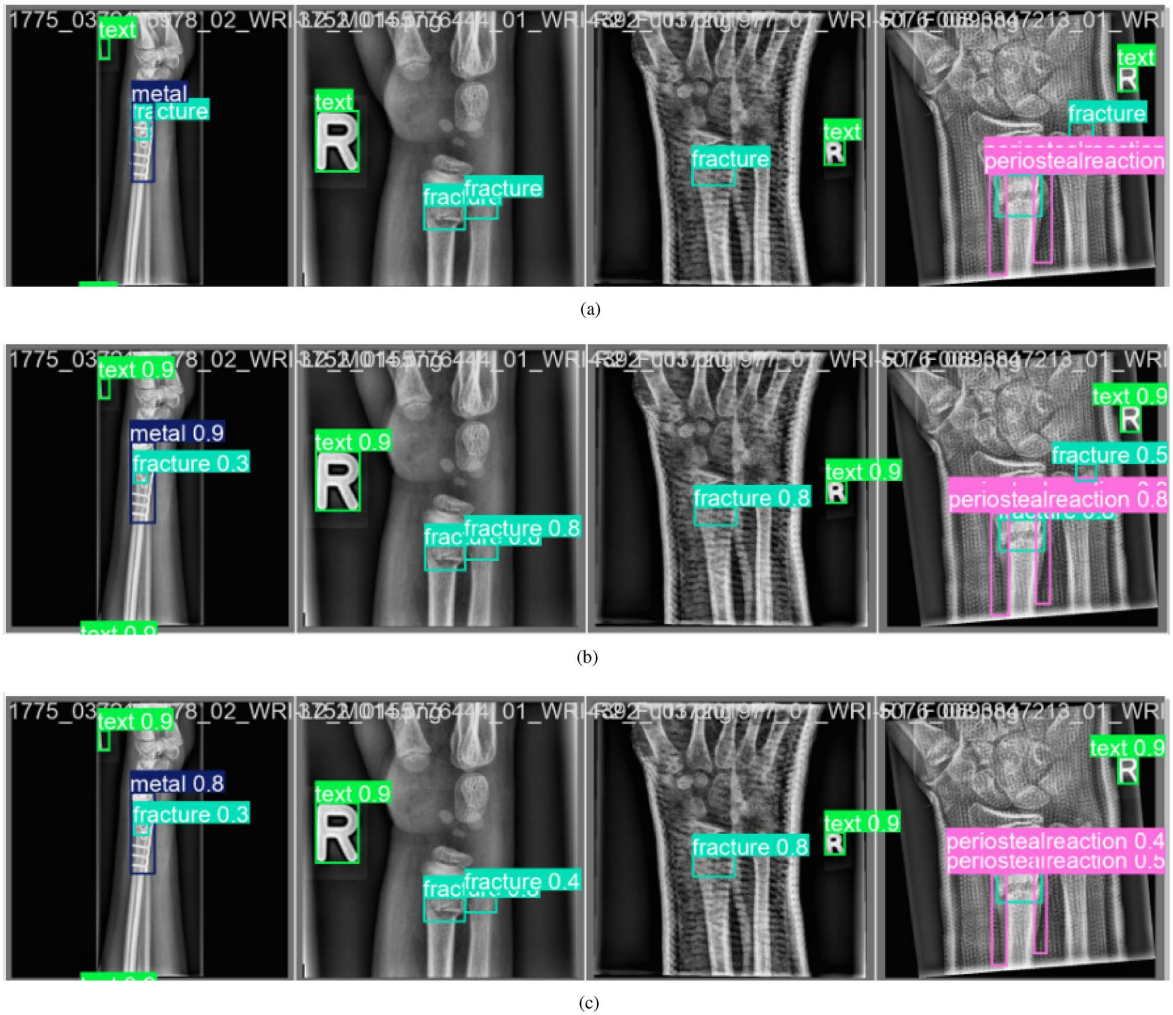
Label comparison of ResYOLOx with YOLO11x.

Figure 5 illustrates sample outputs comparing ResYOLO11x predictions with the ground truth annotations andYOLO11x predictions.

The actual labels from the dataset is shown in the Fig. 5a with proper bounding boxes. The predicted result of our ResYOLO11x model is displayed in Fig. 5b, where the confidence scores for fractures and different abnormalities are shown. The predicted result of the YOLO11x model is displayed in Fig. 5c with corresponding confidence scores for fractures and other different abnormalities.

**Fig. 6:**
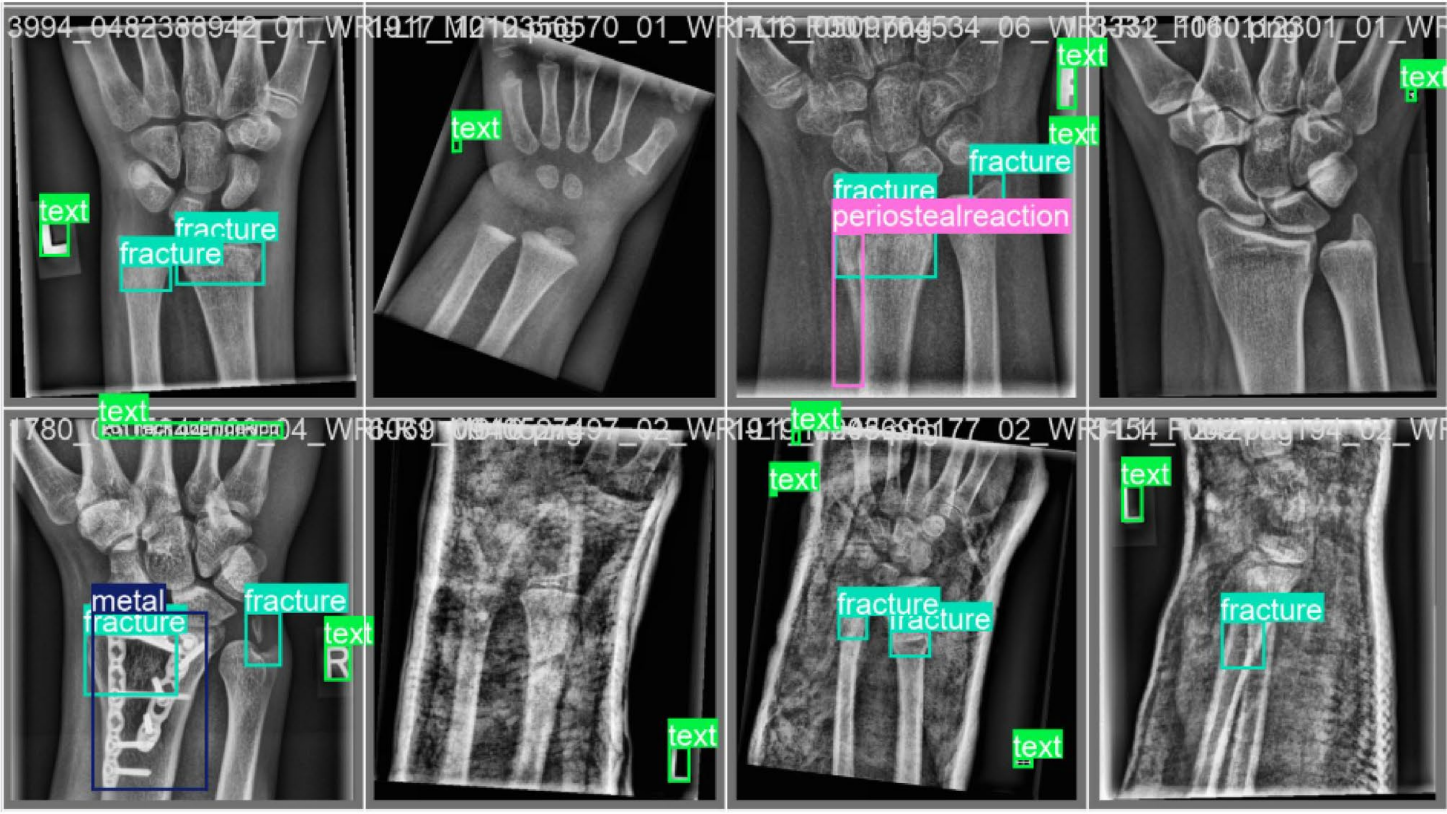
Labeled Images.

**Fig. 7:**
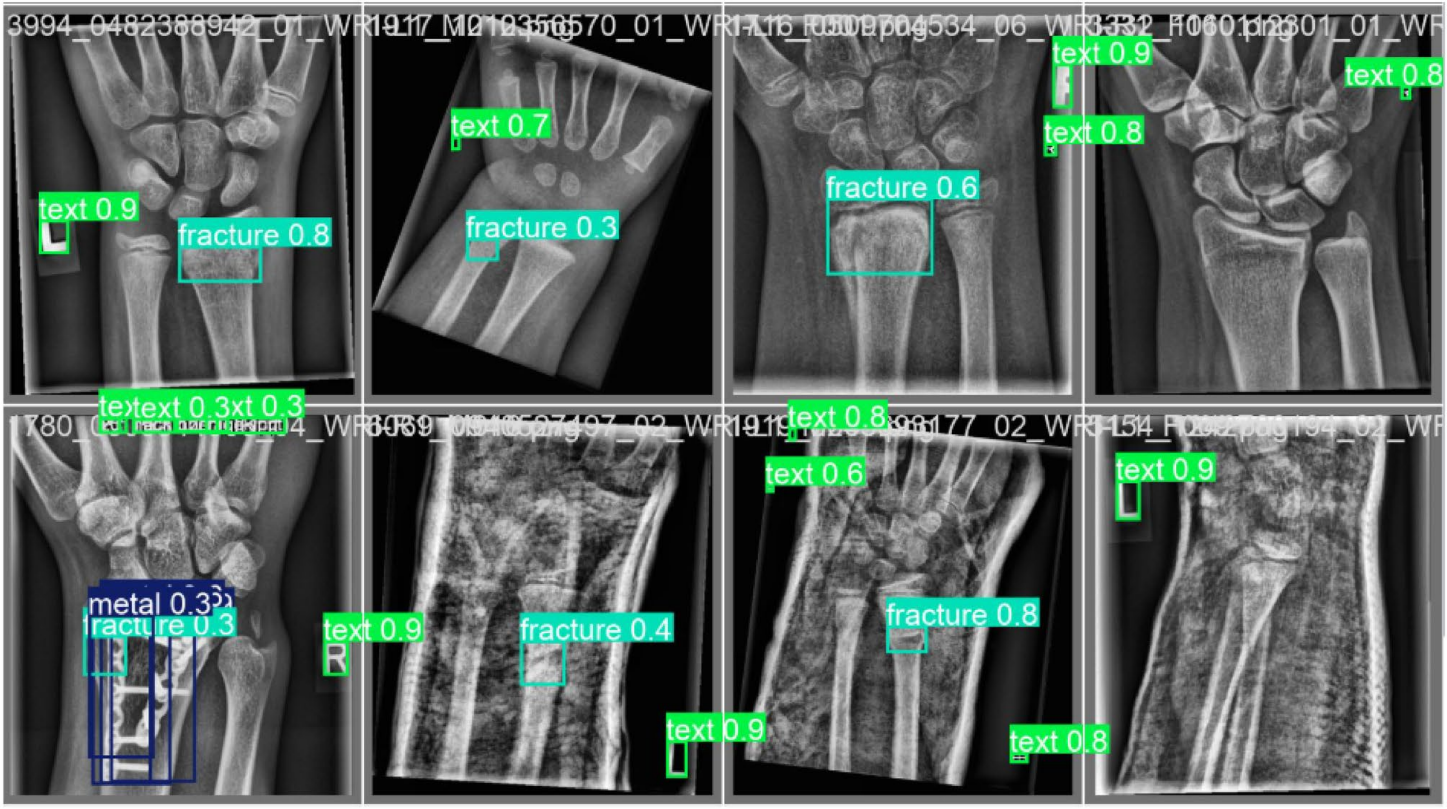
Predicted Images.

The visual comparison validates the enhanced detection capability of the proposed model, particularly in complex and low-contrast cases. Residual feature propagation ensures that subtle discontinuities in bone structure are effectively captured in feature maps, leading to improved bounding box alignment and classification reliability. We also observe that for the same classes, the confidence score for predicting the abnormality or fracture is higher in our proposed ResYOLO11 model. The Labeled Images and Predicted Images are shown in Fig. 6 and Fig. 7 respectively.

### Computational efficiency

In addition to improved detection capability, ResYOLO11 displayed higher computational efficiency. Table 11 provides a detailed comparison of architectural parameters, including layer count, total parameters, GFLOPs, model size, and inference time.

Although the integration of ResNet modules have increased the layers and complexity of the smaller variants of the architecture, the models still remains lighter and faster in the higher size variants. For example, the ResYOLO11x model contains 51.0M parameters and occupies 102.6 MB, compared to 56.8M parameters and 228.3 MB for YOLO11x, representing approx 50% reduction in model size. Moreover, inference time decreased from 0.034 s to 0.026 s per image, confirming the model’s suitability for real-time diagnostic use.

This demonstrates that ResYOLO11 achieves a favorable balance between detection accuracy and computational cost, a critical consideration for deployment in medical imaging environments with limited hardware resources.

**Table 11:** Comparison of ResYOLO11 and YOLO11 models in terms of layers, parameters, gradients, GFLOPS, model size, and inference time.

**Models**	**Layers**	**Parameters**	**Gradients**	**GFLOPS**	**Model Size**	**Inference Time**
ResYOLO11n	388	10,514,507	10,514,491	49.9	21.37 MB	0.012
YOLO11n	319	2,591,595	2,591,579	6.4	5.44 MB	0.009
ResYOLO11s	388	17,995,371	17,995,355	60.1	36.34 MB	0.014
YOLO11s	319	9,431,275	9,431,259	21.6	19.15 MB	0.010
ResYOLO11m	442	23,746,603	23,746,587	79.1	47.9 MB	0.016
YOLO11m	409	20,059,947	20,059,931	68.2	40.5 MB	0.013
ResYOLO11l	589	27,841,323	27,841,307	89.1	56.21 MB	0.019
YOLO11l	631	25,317,419	25,317,403	87.3	51.17 MB	0.017
ResYOLO11x	589	51,007,787	51,007,771	140.4	102.59 MB	0.026
YOLO11x	631	56,884,171	56,884,155	195.5	228.36 MB	0.034

.

### Generalization

For generalization of the model findings, another dataset that is used. Training and evaluation of the models in the FracAtlas dataset^47^. was conducted using the same parameters on the pretrained ResYOLO11 models. The FracAtlas dataset consists of more than 4000 X-ray images of which more than 800 are fractured and the rest are unfractured. The dataset consists of different body parts such as hand, leg, hip, shoulder, etc. The images are all annotated in COCO, VGG, YOLO and Pascal Voc formats.

For the training purpose the dataset is augmented using shearing, $\pm ~20^\circ$ angular rotation, and $90^\circ$ clockwise and anti-clockwise flip. After augmentation the total number of images were 9457 which after 3 fold cross validation were split into sets of 3153, 3152, and 3152 images.

As shown in Table 12, ResYOLO11 consistently outperforms the corresponding YOLO11 baseline across all model scales on the FracAtlas dataset, demonstrating improved fracture detection performance beyond the wrist-specific training domain. The observed gains in F1 score, precision, and mAP@50 indicate that the proposed residual feature integration enhances representation robustness across different anatomical regions. These results confirm the cross-dataset generalization capability of ResYOLO11 without requiring architecture-specific tuning.

**Table 12:** Performance comparison of ResYOLO11 and YOLO11 variants on the FracAtlas dataset across different model scales.

Scale	**ResYOLO11**					**YOLO11**				
	**F1**	**Pre**	**Rec**	**mAP50**	**mAP50-95**	**F1**	**Pre**	**Rec**	**mAP50**	**mAP50-95**
n	0.946	0.961	0.931	0.957	0.763	0.921	0.960	0.885	0.937	0.850
s	0.956	0.976	0.936	0.960	0.789	0.937	0.961	0.915	0.945	0.715
m	0.954	0.968	0.940	0.963	0.788	0.932	0.947	0.918	0.955	0.735
l	0.944	0.967	0.922	0.961	0.767	0.937	0.957	0.918	0.956	0.719
x	0.958	0.971	0.946	0.966	0.794	0.937	0.942	0.932	0.959	0.752

### Clinical applicability and ethical considerations

The proposed ResYOLO11 model is intended to support fracture detection as part of a computer-aided diagnostic workflow rather than replace specialists and function as an autonomous decision-making system. Because of its single-stage design and low inference latency, the proposed model is suitable for clinical environments where fast assessment of radiographic images is required, such as emergency departments and orthopedic clinics. By localizing potential fracture regions, the system can assist clinicians in reviewing images more efficiently, particularly in high-volume settings, while preserving final diagnostic responsibility with medical professionals. The reduced model size also allows deployment on commonly available clinical hardware without requiring substantial changes to existing radiology workflows.

The use of a pediatric wrist-specific dataset introduces inherent limitations related to anatomical bias, and the results should therefore be interpreted within this context. Direct generalization to adult populations or fractures at other anatomical sites may require additional validation. While the model provides visual localization of detected abnormalities, its outputs are intended to be used in conjunction with clinical expertise. Furthermore, the clinical implications of misclassification must be carefully considered. False-positive detections may lead to unnecessary follow-up imaging or additional clinical review, potentially increasing workload and patient anxiety, although such errors are generally less critical than missed diagnoses. In contrast, false negatives pose a higher clinical risk, as undetected fractures may delay treatment and adversely affect patient outcomes, particularly in pediatric cases where fracture patterns can be subtle.

All data used in this study were obtained from publicly available, anonymized sources, and no direct patient interaction was involved. Future clinical deployment would require appropriate ethical approval and adherence to relevant data protection and medical governance standards.

## Conclusion

The paper proposed an architecture (ResYOLO11) for automated, efficient and precise bone fracture detection. The proposed architecture integrates the residual feature learning capacity of ResNet with light-weight multi-scale YOLO11 model, thereby enhancing spatial representation and fracture localization accuracy.

Training and evaluating the model using ‘GRAZPEDWRI-DX’ dataset has demonstrated the consistency of ResYOLO11’s performance on all the variants. The model achieved a peak precision of 0.963, recall of 0.958, and mAP@50 and mAP@50–95 scores of 0.986 and 0.598, respectively, in its extra-large configuration. Notably, ResYOLO11x achieved these results while reducing model size by approximately 50% and inference time by 20%, highlighting its potential suitability for real-time diagnostic applications. It is also observed that the proposed model is highly optimized as compared to the YOLO11 variants of the same size as the number of floating point operations exceeds them.

Compared with existing YOLO based approaches, the proposed architecture achieved higher generalization across various abnormality classes. Thus we can say that the proposed architecture is an effective derivation of the YOLO11 model, that is hybridized with the ResNet layers to achieve better feature extraction, in turn give better detection results. In clinical applicability, the proposed ResYOLO11 model holds promise as a practical CAMD tool for orthopedic and radiological assessment, providing fast and reliable detection support that may reduce diagnostic errors and improve workflow efficiency in emergency and rural healthcare settings.

For future research, training the model in higher resolutions is to be tested. Obtaining optimized hyperparameters, customized for each variant is also a requirement to be satisfied. Further testing of the model in different datasets can also increase its robustness, expanding the scope from only wrist fractures to other body parts. Also, methods to reduce the complexity of the model with the help of other DL-based methods can be integrated into our model. Light-weight deployment is another application that is to be further researched. Integration of eXplanable AI is also another direction to look forward to for enhancing interpretability and trust in the model.

## Supplementary Information

Supplementary Information.

## Data Availability

Data Availability Statement modified as ”The data that support the findings of this study are publicly available in https://doi.org/10.1038/s41597-022-01328-z, https://doi.org/10.1371/journal.pone.0265949, and https://doi.org/10.1016/j.ndteint.2024.103051.”

## References

[1] Rafi, T. H., Shubair, R. M., Farhan, F., Hoque, M. Z. & Quayyum, F. M. Recent advances in computer-aided medical diagnosis using machine learning algorithms with optimization techniques. *IEEE Access* **9**, 137847–137868. 10.1109/ACCESS.2021.3108892 (2021).

[2] Yeasmin, M. N., Al Amin, M., Joti, T. J., Aung, Z. & Azim, M. A. Advances of ai in image-based computer-aided diagnosis: A review. *Array*10.1016/j.array.2024.100357 (2024).

[3] Joshi, D. & Singh, T. P. A survey of fracture detection techniques in bone x-ray images. *Artif. Intell. Rev.* **53**, 4475–4517. 10.1007/s10462-019-09799-0 (2020).

[4] Genant, H., Engelke, K. & Prevrhal, S. Advanced CT bone imaging in osteoporosis. *Rheumatology* **47**, iv9–iv16. 10.1093/rheumatology/ken180 (2008). 18556648 10.1093/rheumatology/ken180 PMC2427166

[5] Chang, G. et al. MRI assessment of bone structure and microarchitecture. *J. Magn. Reson. Imaging* **46**, 323–337. 10.1002/jmri.25647 (2017). 28165650 10.1002/jmri.25647 PMC5690546

[6] Singaram, S. & Naidoo, M. The physical, psychological and social impact of long bone fractures on adults: A review. *Afr. J. Prim. Health Care Fam. Med.* **11**, 1–9 (2019). 10.4102/phcfm.v11i1.1908 PMC6556928 31170796

[7] Hedström, E. M., Svensson, O., Bergström, U. & Michno, P. Epidemiology of fractures in children and adolescents: Increased incidence over the past decade: A population-based study from northern sweden. *Acta Orthop.* **81**, 148–153. 10.3109/17453671003628780 (2010). 20175744 10.3109/17453671003628780 PMC2856220

[8] Lin, T.-Y., Goyal, P., Girshick, R., Kaiming, H. & Dollár, P. Focal loss for dense object detection. In *Proceedings of the IEEE International Conference on Computer Vision (ICCV)*, 2980–2988 (2017).

[9] Law, H. & Deng, J. Cornernet: Detecting objects as paired keypoints. In *Proceedings of the European Conference on Computer Vision (ECCV)*, 734–750 (2018).

[10] Zhou, T. et al. Parallel attention multi-scale mandibular fracture detection network based on centernet. *Biomed. Signal Process. Control* **95**, 106338 (2024).

[11] Liu, W. et al. Ssd: Single shot multibox detector. In *Computer Vision–ECCV 2016: 14th European Conference, Amsterdam, The Netherlands, October 11–14, 2016, Proceedings, Part I, 21–37* (Springer International Publishing, 2016).

[12] Liu, S. & Zhang, H. Parallelnet: A depth-guided parallel convolutional network for scene segmentation. In PRICAI 2018: Trends in Artificial Intelligence: 15th Pacific Rim International Conference on Artificial Intelligence, Nanjing, China, August 28–31, 2018, Proceedings, Part I, 588–603 (Springer International Publishing, 2018).

[13] Su, Z., Adam, A., Nasrudin, M. F., Ayob, M. & Punganan, G. Skeletal fracture detection with deep learning: A comprehensive review. *Diagnostics* **13**, 3245. 10.3390/diagnostics13203245 (2023). 37892066 10.3390/diagnostics13203245 PMC10606060

[14] Abbas, W. et al. Lower leg bone fracture detection and classification using faster rcnn for x-rays images. In *2020 IEEE 23rd International Multitopic Conference (INMIC)*, 1–6, 2020, 10.1109/INMIC50486.2020.9318052 (IEEE, 2020).

[15] Giełczyk, A., Marciniak, A., Tarczewska, M. & Lutowski, Z. Pre-processing methods in chest x-ray image classification. *Plos one* **17**, e0265949. 10.1371/journal.pone.0265949 (2022). 35381050 10.1371/journal.pone.0265949 PMC8982897

[16] Wu, Y. et al. An enhancement algorithm based on multi-grayscale fusion and edge-weight for low contrast x-ray image. *NDT & E Int.* **143**, 103051. 10.1016/j.ndteint.2024.103051 (2024).

[17] Ahamed, K. U. et al. A deep learning approach using effective preprocessing techniques to detect covid-19 from chest CT-scan and x-ray images. *Comput. Biol. Med.* **139**, 105014. 10.1016/j.compbiomed.2021.105014 (2021). 34781234 10.1016/j.compbiomed.2021.105014 PMC8566098

[18] Xiao, L., Li, C., Wu, Z. & Wang, T. An enhancement method for x-ray image via fuzzy noise removal and homomorphic filtering. *Neurocomputing* **195**, 56–64. 10.1016/j.neucom.2015.08.113 (2016).

[19] Juneja, M., Minhas, J. S., Singla, N., Kaur, R. & Jindal, P. Denoising techniques for cephalometric x-ray images: A comprehensive review. *Multimed. Tools Appl.* **83**, 49953–49991. 10.1007/s11042-023-17495-z (2024).

[20] Gonzalez, R. C. *Digital Image Processing* (Pearson Education India, 2009).

[21] Michel-González, E., Cho, M. H. & Lee, S. Y. Geometric nonlinear diffusion filter and its application to x-ray imaging. *Biomed. Eng. Online* **10**, 1–16. 10.1186/1475-925X-10-47 (2011). 21639933 10.1186/1475-925X-10-47 PMC3121643

[22] Öktem, H., Egiazarian, K., Niittylahti, J. & Lemmetti, J. An approach to adaptive enhancement of diagnostic x-ray images. *EURASIP J. Adv. Signal Process.* **1–7**, 2003. 10.1155/S1110865703211069 (2003).

[23] Malarvel, M. et al. Anisotropic diffusion based denoising on x-radiography images to detect weld defects. *Digit. Signal Process.* **68**, 112–126. 10.1016/j.dsp.2017.05.014 (2017).

[24] He, K., Zhang, X., Ren, S. & Sun, J. Deep residual learning for image recognition. In *Proceedings of the IEEE Conference on Computer Vision and Pattern Recognition*, 770–778 (2016).

[25] Park, S. A guide to two-stage object detection: R-CNN, FPN, mask R-CNN. *medium. com* (2021).

[26] Bacea, D.-S. & Oniga, F. Single stage architecture for improved accuracy real-time object detection on mobile devices. *Image Vis. Comput.* **130**, 104613. 10.1016/j.imavis.2022.104613 (2023).

[27] Ma, Y. & Luo, Y. Bone fracture detection through the two-stage system of crack-sensitive convolutional neural network. *Inform. Med. Unlocked* **22**, 100452. 10.1016/j.imu.2020.100452 (2021).

[28] El-Saadawy, H., Tantawi, M., Shedeed, H. A. & Tolba, M. F. One-stage vs two-stage deep learning method for bone abnormality detection. In *The International Conference on Artificial Intelligence and Computer Vision*, 122–132, (2021), 10.1007/978-3-030-76346-6_12 (Springer, 2021).

[29] Zeng, B. et al. Two-stage structure-focused contrastive learning for automatic identification and localization of complex pelvic fractures. *IEEE Trans. Med. Imaging* **42**, 2751–2762. 10.1109/TMI.2023.3264298 (2023). 37030821 10.1109/TMI.2023.3264298

[30] Yaseen, M. et al. Cervical spine fracture detection and classification using two-stage deep learning methodology. *IEEE Access* **12**, 72131–72142. 10.1109/ACCESS.2024.3398061 (2024).

[31] Zhang, Y., Li, X., Wang, F., Wei, B. & Li, L. A comprehensive review of one-stage networks for object detection. In *2021 IEEE International Conference on Signal Processing, Communications and Computing* (ICSPCC), 1–6, (2021), 10.1109/ICSPCC52875.2021.9564613 (IEEE, 2021).

[32] Zou, J. & Arshad, M. R. Detection of whole body bone fractures based on improved yolov7. *Biomed. Signal Process. Control* **91**, 105995. 10.1016/j.bspc.2024.105995 (2024).

[33] Inui, A. et al. Detection of elbow OCD in the ultrasound image by artificial intelligence using yolov8. *Appl. Sci.* **13**, 7623. 10.3390/app13137623 (2023).

[34] Nagy, E., Janisch, M., Hržić, F., Sorantin, E. & Tschauner, S. A pediatric wrist trauma x-ray dataset (GRAZPEDWRI-DX) for machine learning. *Sci. Data* **9**, 222. 10.1038/s41597-022-01328-z (2022). 35595759 10.1038/s41597-022-01328-z PMC9122976

[35] Nguyen, H. T., Tran, T. B. & Tran, T. T. Fracture detection in bone: An approach with versions of yolov4. *SN Comput. Sci.* **5**, 765. 10.1007/s42979-024-03155-y (2024).

[36] Ahmed, A., Imran, A. S., Manaf, A., Kastrati, Z. & Daudpota, S. M. Enhancing wrist abnormality detection with yolo: Analysis of state-of-the-art single-stage detection models. *Biomed. Signal Process. Control* **93**, 106144. 10.1016/j.bspc.2024.106144 (2024).

[37] Chien, C.-T., Ju, R.-Y., Chou, K.-Y. & Chiang, J.-S. Yolov9 for fracture detection in pediatric wrist trauma x-ray images. *Electron. Lett.* **60**, e13248. 10.1049/ell2.13248 (2024).

[38] Wu, J. End-to-end pediatric bone fracture localization with data augmentation.

[39] Ahmed, A. & Manaf, A. Pediatric wrist fracture detection in x-rays via yolov10 algorithm and dual label assignment system. arXiv preprint arXiv:2407.15689https://doi.org/10.48550/arXiv.2407.15689 (2024)

[40] Wang, H., Li, Z. & Zhang, D. A two-stage deep learning method for auxiliary diagnosis of upper limb fractures based on ResNet-50 and enhanced yolo. *Mathematics*10.3390/math13111858 (2025).40855850

[41] Medaramatla, S. C., Samhitha, C. V., Pande, S. D. & Vinta, S. R. Detection of hand bone fractures in x-ray images using hybrid YOLO NAS. *IEEE Access* **12**, 57661–57673. 10.1109/ACCESS.2024.3379760 (2024).

[42] Alam, T. et al. An integrated approach using yolov8 and ResNet, SERESNET & vision transformer (vit) algorithms based on ROI fracture prediction in x-ray images of the elbow. *Curr. Med. Imaging* **20**, e15734056309890. 10.2174/0115734056309890240912054616 (2024). 39360542 10.2174/0115734056309890240912054616

[43] Rao, M. G. et al. Enhancing fracture detection in different bones using deep learning and yolo frameworks. In *2025 3rd International Conference on Smart Systems for applications in Electrical Sciences (ICSSES)*, (2025), 10.1109/ICSSES64899.2025.11009941.

[44] Liu, B. DW-YOLO: improved YOLO for bone fracture detection. In: Lei, T. & Zhang, D. (eds.) *Sixth International Conference on Information Science, Electrical, and Automation Engineering (ISEAE 2024)*, vol. 13275, 132751T, 10.1117/12.3037465. International Society for Optics and Photonics (SPIE, 2024).

[45] Taş, H. G., Taş, M. B. H., Yildiz, E. & Aydin, S. Early detection of lung metastases in breast cancer using yolov10 and transfer learning: A diagnostic accuracy study. Medical science monitor. *Int. Med. J. Exp. Clin. Res.* **31**, e948195. 10.12659/MSM.948195 (2025). 10.12659/MSM.948195 PMC12433176 40922404

[46] Jocher, G. & Qiu, J. *Ultralytics YOLO11* (San Francisco, CA, USA, GitHub, 2024).

[47] Abedeen, I. et al. Fracatlas: A dataset for fracture classification, localization and segmentation of musculoskeletal radiographs. *Sci. Data* **10**, 521. 10.1038/s41597-023-02432-4 (2023). 37543626 10.1038/s41597-023-02432-4 PMC10404222

