# Supplementary material for: ResNet based backbone integrated YOLO framework for bone fracture detection
Source: Sci Rep. 2026 Mar 10;16:12954. doi: 10.1038/s41598-026-41782-y (PMC13096436; doi:10.1038/s41598-026-41782-y)
Supplement: Supplementary file 1 — Supplementary Information. [file 41598_2026_41782_MOESM1_ESM.docx]

Supplementary material:

Table on Standard Deviation in Precision (Table 7)

|  | **ResYOLO11** | | | | |  | **YOLO11** | | | | |
| --- | --- | --- | --- | --- | --- | --- | --- | --- | --- | --- | --- |
| **Class** | **n** | **s** | **m** | **l** | **x** |  | **n** | **s** | **m** | **l** | **x** |
| **all** | 0.021 | 0.02 | 0.016 | 0.066 | 0.019 |  | 0.036 | 0.047 | 0.049 | 0.08 | 0.007 |
| **boneanomaly** | 0.067 | 0.023 | 0.048 | 0.107 | 0.004 |  | 0.091 | 0.038 | 0.061 | 0.136 | 0.041 |
| **bonelession** | 0.233 | 0.131 | 0.111 | 0.03 | 0.082 |  | 0.328 | 0.43 | 0.328 | 0.234 | 0.026 |
| **foreignbody** | 0 | 0 | 0 | 0 | 0 |  | 0 | 0.021 | 0 | 0.117 | 0 |
| **fracture** | 0.005 | 0.003 | 0.008 | 0.036 | 0.011 |  | 0.021 | 0.015 | 0.012 | 0.027 | 0.009 |
| **metal** | 0.008 | 0.002 | 0.017 | 0.046 | 0.007 |  | 0.024 | 0.085 | 0.029 | 0.029 | 0.013 |
| **periostealreaction** | 0.028 | 0.032 | 0.02 | 0.12 | 0.036 |  | 0.076 | 0.001 | 0.02 | 0.13 | 0.018 |
| **pronatorsign** | 0.003 | 0.019 | 0.032 | 0.092 | 0.043 |  | 0.078 | 0.035 | 0.024 | 0.1 | 0.012 |
| **softtissue** | 0.079 | 0.038 | 0.045 | 0.147 | 0.055 |  | 0.146 | 0.196 | 0.035 | 0.085 | 0.046 |
| **text** | 0.003 | 0.003 | 0.003 | 0.016 | 0.002 |  | 0.007 | 0.001 | 0.002 | 0.012 | 0.001 |

Table on Standard Deviation in Recall (Table 8)

|  | **ResYOLO11** | | | | |  | **YOLO11** | | | | |
| --- | --- | --- | --- | --- | --- | --- | --- | --- | --- | --- | --- |
| **Class** | **n** | **s** | **m** | **l** | **x** |  | **n** | **s** | **m** | **l** | **x** |
| **all** | 0.037 | 0.012 | 0.022 | 0.068 | 0.015 |  | 0.039 | 0.079 | 0.051 | 0.132 | 0.027 |
| **boneanomaly** | 0.036 | 0.062 | 0.103 | 0.045 | 0.124 |  | 0.097 | 0.058 | 0.069 | 0.08 | 0.173 |
| **bonelession** | 0.193 | 0.085 | 0.072 | 0.054 | 0.043 |  | 0 | 0.344 | 0.236 | 0.256 | 0.062 |
| **foreignbody** | 0 | 0 | 0 | 0.286 | 0 |  | 0 | 0.555 | 0 | 0.577 | 0 |
| **fracture** | 0.006 | 0.003 | 0.002 | 0.016 | 0.005 |  | 0.01 | 0.017 | 0.016 | 0.023 | 0.016 |
| **metal** | 0.016 | 0.011 | 0.003 | 0.006 | 0.008 |  | 0.011 | 0.171 | 0.003 | 0.004 | 0.014 |
| **periostealreaction** | 0.029 | 0.003 | 0.042 | 0.083 | 0.042 |  | 0.06 | 0.092 | 0.029 | 0.084 | 0.05 |
| **pronatorsign** | 0.068 | 0.041 | 0.054 | 0.12 | 0.051 |  | 0.095 | 0.149 | 0.036 | 0.109 | 0.035 |
| **softtissue** | 0.086 | 0.036 | 0.038 | 0.143 | 0.019 |  | 0.108 | 0.27 | 0.086 | 0.14 | 0.061 |
| **text** | 0.002 | 0.002 | 0.004 | 0.002 | 0.005 |  | 0.004 | 0.004 | 0.002 | 0.004 | 0.002 |

Table on Standard Deviation in mAP50 (Table 9)

|  | **ResYOLO11** | | | | |  | **YOLO11** | | | | |
| --- | --- | --- | --- | --- | --- | --- | --- | --- | --- | --- | --- |
| **Class** | **n** | **s** | **m** | **l** | **x** |  | **n** | **s** | **m** | **l** | **x** |
| **all** | 0.016 | 0.014 | 0.002 | 0.031 | 0.038 |  | 0.007 | 0.045 | 0.024 | 0.037 | 0.021 |
| **boneanomaly** | 0.017 | 0.031 | 0.097 | 0.106 | 0.096 |  | 0.07 | 0.074 | 0.099 | 0.045 | 0.169 |
| **bonelession** | 0.076 | 0.051 | 0.006 | 0.025 | 0.059 |  | 0.053 | 0.329 | 0.26 | 0.251 | 0.081 |
| **foreignbody** | 0.086 | 0.061 | 0.103 | 0.274 | 0.249 |  | 0.146 | 0.489 | 0.288 | 0.33 | 0.086 |
| **fracture** | 0.003 | 0.001 | 0.001 | 0.003 | 0.005 |  | 0.002 | 0.008 | 0.008 | 0.009 | 0.001 |
| **metal** | 0.009 | 0.002 | 0.005 | 0.004 | 0.004 |  | 0.013 | 0.112 | 0.006 | 0.005 | 0.002 |
| **periostealreaction** | 0.008 | 0.017 | 0.018 | 0.02 | 0.012 |  | 0.018 | 0.072 | 0.045 | 0.04 | 0.044 |
| **pronatorsign** | 0.019 | 0.051 | 0.05 | 0.043 | 0.047 |  | 0.01 | 0.101 | 0.021 | 0.024 | 0.018 |
| **softtissue** | 0.041 | 0.018 | 0.019 | 0.004 | 0.031 |  | 0.008 | 0.231 | 0.077 | 0.058 | 0.076 |
| **text** | 0.001 | 0.001 | 0.001 | 0 | 0.001 |  | 0.001 | 0.003 | 0.001 | 0.001 | 0.001 |

Table on Standard Deviation in mAP50-95 (Table 10)

|  | **ResYOLO11** | | | | |  | **YOLO11** | | | | |
| --- | --- | --- | --- | --- | --- | --- | --- | --- | --- | --- | --- |
| **Class** | **n** | **s** | **m** | **l** | **x** |  | **n** | **s** | **m** | **l** | **x** |
| **all** | 0.018 | 0.015 | 0.015 | 0.023 | 0.032 |  | 0.009 | 0.041 | 0.026 | 0.02 | 0.02 |
| **boneanomaly** | 0.025 | 0.035 | 0.063 | 0.047 | 0.078 |  | 0.04 | 0.031 | 0.107 | 0.054 | 0.143 |
| **bonelession** | 0.079 | 0.06 | 0.051 | 0.04 | 0.041 |  | 0.011 | 0.267 | 0.198 | 0.165 | 0.056 |
| **foreignbody** | 0.077 | 0.013 | 0.1 | 0.203 | 0.174 |  | 0.132 | 0.364 | 0.29 | 0.264 | 0.074 |
| **fracture** | 0.004 | 0.002 | 0.003 | 0.002 | 0.001 |  | 0.007 | 0.077 | 0.041 | 0.032 | 0.038 |
| **metal** | 0.014 | 0.009 | 0.006 | 0.012 | 0.011 |  | 0.004 | 0.2 | 0.015 | 0.01 | 0.013 |
| **periostealreaction** | 0.015 | 0.008 | 0.005 | 0.008 | 0.003 |  | 0.019 | 0.095 | 0.051 | 0.033 | 0.047 |
| **pronatorsign** | 0.012 | 0.041 | 0.038 | 0.038 | 0.038 |  | 0.011 | 0.069 | 0.037 | 0.026 | 0.026 |
| **softtissue** | 0.035 | 0.033 | 0.026 | 0.012 | 0.038 |  | 0.008 | 0.211 | 0.066 | 0.042 | 0.061 |
| **text** | 0.002 | 0.004 | 0.004 | 0.003 | 0.004 |  | 0.001 | 0.006 | 0.006 | 0.007 | 0.004 |
